# Bridging Oral Health and Integrated Care: A Scientometric Study of Integrated Care for Older People (ICOPE) and Removable Prosthodontics in Older Adults

**DOI:** 10.7759/cureus.106875

**Published:** 2026-04-12

**Authors:** Panagiota Chatzidou, John Fanourgiakis, Vassiliki Anastassiadou

**Affiliations:** 1 Department of Prosthodontics, Aristotle University of Thessaloniki, Thessaloniki, GRC; 2 Department of Management Science and Technology, Hellenic Mediterranean University‎, Heraklion, GRC; 3 Department of Healthcare Management, Faculty of Social Sciences, Hellenic Open University, Patras, GRC; 4 Department of Prosthodontics, School of Dentistry, Aristotle University of Thessaloniki, Thessaloniki, GRC

**Keywords:** frailty, icope, intrinsic capacity, oral health, prosthodontics

## Abstract

Population aging has shifted healthcare priorities from disease-centered models towards functional ability and quality of life. The WHO Integrated Care for Older People (ICOPE) framework operationalizes healthy aging through assessment of intrinsic capacity (IC), yet oral health and prosthodontic rehabilitation remain underrepresented. This study aimed to map and analyze the scientific literature linking ICOPE with removable prosthodontics, focusing on intrinsic capacity, oral function, and functional outcomes in older adults. A bibliometric search was conducted in Scopus (ICOPE OR "Integrated Care for Older People" OR "WHO ICOPE" OR "intrinsic capacity") AND ("removable prosthodontics" OR "removable dental prostheses" OR dentures OR "removable partial denture*" OR "complete denture*") AND (PUBYEAR >2016 ) AND PUBYEAR <2027 AND (LIMIT-TO {SUBJAREA, "MEDI"} OR LIMIT-TO {SUBJAREA, "NURS"} OR LIMIT-TO {SUBJAREA, "DENT"} OR LIMIT-TO {SUBJAREA, "HEAL"}) AND (LIMIT-TO {DOCTYPE, "ar"} OR LIMIT-TO {DOCTYPE, "re"}) AND (LIMIT-TO {LANGUAGE, "English"}), limited to January 31, 2026, and restricted to English-language peer-reviewed articles in medical, nursing, dental, and health sciences. Data extracted covered study characteristics, participant demographics, thematic focus, and citation metrics, with studies classified by design. Thematic focus included IC assessment, oral function/prosthodontics, nutrition/frailty, and quality of life. Coauthorship and term clustering analyses were performed using VOSviewer (Leiden, The Netherlands: Centre for Science and Technology Studies, Leiden University) to visualize collaborative and conceptual networks. Thirteen studies were identified. European studies emphasized clinical assessment, prosthodontics, and policy; Asian studies focused on malnutrition, IC transitions, and functional decline. The following four clusters emerged: (1) ICOPE and IC; (2) oral function and frailty; (3) removable prosthodontics and quality of life; (4) cognitive and psychosocial outcomes. Oral hypofunction and tooth loss were associated with IC decline, frailty, and HRQoL, whereas prosthodontics improved masticatory function, nutrition, psychosocial well-being, and cognition. Integrating oral health and removable prosthodontics into ICOPE frameworks may enhance early detection of functional decline and promote healthy aging.

## Introduction and background

Population aging is accelerating worldwide, prompting a shift from disease-centered models of care towards approaches that prioritize functional ability and quality of life in later life. In response to this demographic transition, the World Health Organization (WHO) introduced the healthy aging framework, defining healthy aging as the process of developing and maintaining functional ability that enables well-being in older age [1]. Central to this framework is the concept of intrinsic capacity, which refers to the composite of an individual’s physical and mental capacities and underpins functional ability across the life course [2].

To operationalize this framework in clinical and community settings, the World Health Organization (WHO) developed the Integrated Care for Older People (ICOPE) model. ICOPE provides a person-centered, integrated approach to assessing and managing declines in intrinsic capacity, focusing on the following five core domains: locomotion, cognition, vitality (nutrition), sensory capacity, and psychological well-being [3-5]. Since its introduction, ICOPE has increasingly guided aging-related research, primary care pathways, and health system reforms globally.

Despite its comprehensive scope, oral health is not explicitly included as a core domain within the ICOPE framework. This omission is notable given the well-established role of oral function in supporting nutrition, communication, social participation, and overall quality of life in older adults. Tooth loss, impaired mastication, and reduced oral function are prevalent in aging populations and are strongly associated with frailty, malnutrition, and functional decline [6].

Emerging evidence indicates that oral health and oral function are closely linked to intrinsic capacity. Recent studies have shown significant associations between oral hypofunction and declines in intrinsic capacity domains, including vitality, mobility, and cognition [7]. These findings position oral function as a potential modifiable determinant of intrinsic capacity, aligning with ICOPE’s preventive and functional focus.

Removable prosthodontic rehabilitation, including complete and removable partial dentures, plays a critical role in restoring oral function in older adults. Prosthetic treatment has been shown to improve masticatory performance, nutritional intake, oral health-related quality of life, and psychosocial well-being [6]. Moreover, growing evidence suggests that prosthetic rehabilitation may influence broader health outcomes, including cognitive function, further reinforcing its relevance to intrinsic capacity domains [8].

However, the extent to which removable prosthodontics is conceptually or empirically integrated into the ICOPE literature remains unclear. To date, no scientometric study has systematically examined the intersection of ICOPE, intrinsic capacity, and removable prosthodontic care. Understanding how oral health and prosthodontics are represented in the evolving ICOPE research landscape is essential for identifying knowledge gaps and informing the development of more comprehensive, interdisciplinary models of healthy aging.

Therefore, this scientometric study aimed to map and analyze the scientific literature linking the WHO ICOPE framework with removable prosthodontics, with particular emphasis on intrinsic capacity, oral function, and functional outcomes in older adults. By elucidating publication trends, thematic structures, and research gaps, this study seeks to highlight the potential role of removable prosthodontics as an integral component of integrated care for older adults.

## Review

Materials and methods

Search Strategy

A bibliometric search was conducted using Scopus to identify publications linking the WHO ICOPE framework, intrinsic capacity, and removable prosthodontics. The search algorithm was as follows: (ICOPE OR "Integrated Care for Older People" OR "WHO ICOPE" OR "intrinsic capacity") AND ("removable prosthodontics" OR "removable dental prostheses" OR dentures OR "removable partial denture*" OR "complete denture*") AND (PUBYEAR >2016) AND PUBYEAR <2027 AND (LIMIT-TO {SUBJAREA, "MEDI"} OR LIMIT-TO {SUBJAREA, "NURS"} OR LIMIT-TO {SUBJAREA, "DENT"} OR LIMIT-TO {SUBJAREA, "HEAL"}) AND (LIMIT-TO {DOCTYPE, "ar"} OR LIMIT-TO {DOCTYPE, "re"}) AND (LIMIT-TO {LANGUAGE, "English"}). The search included articles published in the English language up to January 31, 2026.

Inclusion and Exclusion Criteria

Inclusion criteria comprised peer-reviewed articles explicitly addressing intrinsic capacity, ICOPE, or oral health, with a focus on removable prosthodontic interventions in older adults. Exclusion criteria were conference proceedings, book chapters, editorials, commentaries, studies of patient-reported outcome measures unrelated to intrinsic capacity, and studies not involving removable prosthodontics.

Data Extraction

In cases of ties, citation density (number of citations per year) from Scopus was used as the tiebreaker. The following extracted bibliometric data were included: study type (article or review), document title, journal, publication year, authorship, corresponding author's country and continent, study design, sample size, population age, primary clinical focus, thematic focus, number of citations, and citation density.

Study designs were categorized as cross-sectional, cohort, narrative review, systematic review, methodological review, or mixed-methods. Thematic focus was classified into intrinsic capacity assessment, oral function and prosthodontics, nutrition and frailty, and quality of life outcomes.

Network Analysis

Bibliometric networks were generated using VOSviewer 1.6.20 (Leiden, The Netherlands: Centre for Science and Technology Studies, Leiden University), with coauthorship and thematic clustering analyses. Nodes represented authors or key terms, with node size indicating publication frequency and line thickness reflecting connection strength. Clusters were color-coded to illustrate distinct collaborative or conceptual networks, enabling visualization of research trends and thematic relationships.

Results

Study Characteristics and Chronological Trends

A total of 13 studies published between 2018 and 2026 (January 31, 2026) were included in this scientometric study, reflecting the evolving research landscape on oral health, intrinsic capacity, frailty, and healthy aging (Table 1). The earliest study investigated chewing difficulty as a geriatric syndrome, providing foundational insight into oral function and functional decline [9]. Studies published between 2021 and 2022 highlighted growing attention to nutrition, frailty, and longitudinal assessments of intrinsic capacity [10-14]. More recent publications (2023-2026) focused on oral health interventions, long-term care barriers, functional outcomes, and pharmacotherapy [15-21].

**Table 1:** Main information of studies on ICOPE and removable prosthodontics in older adults (n=13). ICOPE: Integrated Care for Older People; LTC: long-term care; IC: intrinsic capacity; HRQoL: health-related quality of life; MNA: Mini Nutritional Assessment; NHANES: National Health and Nutrition Examination Survey

S. no.	Studies	Year	Journal	Study design	Study objective	Sample size	Population age	Corresponding author country	Continent	Main clinical interest	Thematic focus	Exposure window (years)
1	Woo et al. [9]	2018	Nutrients	Population-based cohort	Chewing difficulty and frailty	Large cohort	≥65	Hong Kong	Asia	Geriatric syndrome	Chewing difficulty, frailty	8
2	Patel et al. [10]	2021	Lancet Healthy Longev	Narrative review/policy	Oral health and healthy ageing	NA	Older adults	UK	Europe	Healthy ageing policy	Policy framework	5
3	Ma et al. [11]	2021	J Nutr Health Aging	Narrative review	Frailty in China	NA	Older adults	China	Asia	Frailty implementation	Screening and implementation	5
4	Guigoz and Vellas [12]	2021	J Nutr Health Aging	Narrative review/methodological	MNA history, nutrition, and frailty	NA	Older adults	France	Europe	Nutrition screening, sarcopenia	MNA validation, malnutrition, and frailty	5
5	Khalatbari-Soltani et al. [13]	2022	Int J Epidemiol	Cohort profile	Describe ageing cohort	~1700+ men	≥70	Australia	Oceania	Longitudinal ageing	Frailty and ageing	4
6	Jia et al. [15]	2023	BMC Geriatr	Prospective cohort	IC transitions and disability	Large Chinese cohort	≥60	China	Asia	IC-frailty transitions	IC trajectories	3
7	Chuansangeam et al. [14]	2022	Asia Pac J Clin Nutr	Systematic review and meta-analysis	Malnutrition prevalence and risk	Multiple studies	Older adults	Thailand	Asia	Nutrition and frailty	Malnutrition and risk factors	4
8	Rony et al. [16]	2024	Healthcare	Narrative review	HRQoL determinants	NA	Older adults	Bangladesh	Asia	HRQoL	HRQoL domains	2
9	Sciacchitano et al. [17]	2024	J Clin Med	Narrative review	Frailty models	NA	Older adults	Italy	Europe	Frailty conceptualization	Frailty phenotype vs. deficit model	2
10	Eggimann et al. [18]	2024	Lancet Healthy Longev	Cross-sectional	Oral function comparison	Hospital cohort	Older adults	Switzerland	Europe	Oral hypofunction	Oral function, inpatient vs. outpatient	2
11	Dibello et al. [19]	2025	J Oral Rehabil	Cross-sectional	Tooth loss and IC decline	NHANES ≥60	≥60	Italy	Europe	Tooth loss and IC	Tooth loss, functional decline	1
12	Rani et al. [20]	2025	Healthcare	Mixed-methods	LTC oral care barriers	131	≥60	Malaysia	Asia	LTC oral systems	LTC barriers	1
13	Scuttenaire and Braud [21]	2026	J Clin Exp Dent	Retrospective	Anti-resorptive therapy and oral status	NA	NA	France	Europe	Osteoporosis and oral health	Pharmacotherapy	0-1

Geographic Distribution of Studies

**Figure 1:**
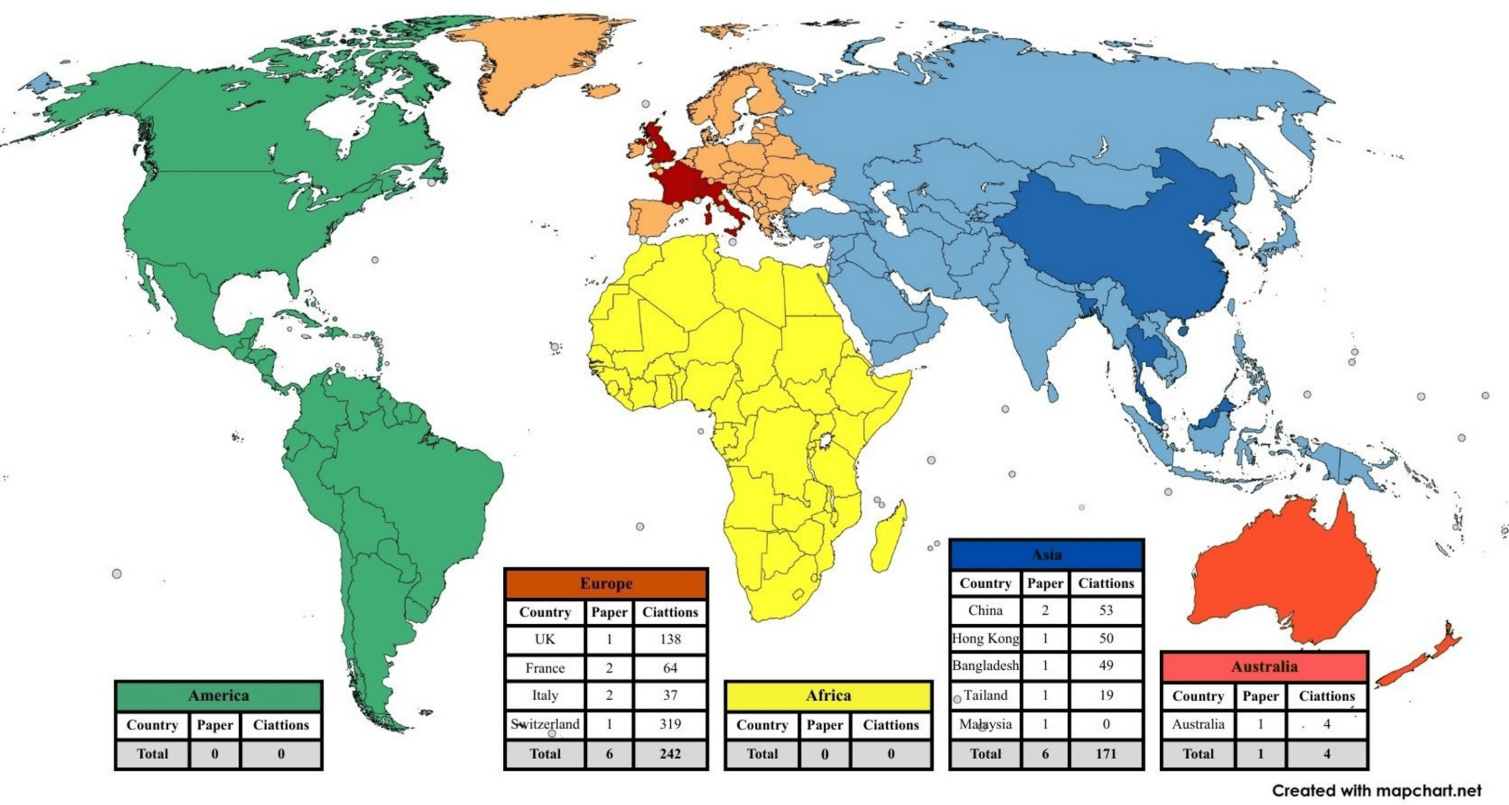
Worldwide distribution of the origin of publications on ICOPE and removable prosthodontics in older adults (n=13). ICOPE: Integrated Care for Older People

The 13 included studies span multiple continents, reflecting both regional and global perspectives on oral health, nutrition, frailty, and intrinsic capacity (IC), with Asia and Europe contributing the majority [9-12,14-21]. Longitudinal cohort data were primarily provided by Khalatbari-Soltani et al. and Jia et al., while cross-sectional and narrative reviews addressed thematic topics such as oral function, frailty, and policy integration [13,15]. Sample sizes ranged from small facility-based cohorts to nationally representative datasets (e.g., National Health and Nutrition Examination Survey {NHANES}, ≥60 years) (Figure 1). In terms of geographical distribution, Europe and Asia demonstrate a balanced level of scientific output, with six publications attributed to each region [9-12,14-21]. Australia was represented by the Concord Health and Ageing in Men Project (CHAMP) cohort study in Australia, providing longitudinal insights into aging men (Figure 1 and Table 2) [13].

**Table 2:** Continent distribution of studies on ICOPE and removable prosthodontics in older adults (n=13). ICOPE: Integrated Care for Older People

Continents	Number of studies (n=13)	Studies	Primary thematic focus
Asia	6	Woo et al. (2018) [9]; Ma et al. (2021) [11]; Chuansangeam et al. (2022) [14]; Jia et al. (2023) [15]; Rony et al. (2024) [16]; Rani et al. (2025) [20]	Intrinsic capacity and frailty transitions; malnutrition; oral hypofunction; functional decline; health-related quality of life (HRQoL) outcomes
Europe	6	Patel et al. (2021) [10]; Guigoz and Vellas (2021) [12]; Sciacchitano et al. (2024) [17]; Eggimann et al. (2024) [18]; Dibello et al. (2025) [19]; Scuttenaire and Braud (2026) [21]	Oral function and prosthodontics; frailty assessment; nutrition screening; policy integration; osteoporosis pharmacotherapy; and oral status in geriatric inpatients
Australia	1	Khalatbari-Soltani et al. (2022) [13]	Longitudinal aging; cohort methodology

This geographic spread shows that research on oral health, frailty, and nutrition is concentrated in Europe and Asia, with fewer contributions from Africa, the Americas, and Australia. European studies tended to focus on clinical assessments, prosthodontics, and policy frameworks, whereas Asian studies emphasized epidemiology, transitions in intrinsic capacity, and the prevalence of malnutrition. This regional variation underscores the need for context-specific strategies and the potential for cross-regional collaborations to strengthen evidence on oral health and functional outcomes in older adults.

The geographic distribution of studies highlights regional research strengths and gaps in the literature linking oral health, intrinsic capacity, and prosthodontics. Europe contributed six studies (38%), primarily focused on clinical assessments, policy integration, frailty evaluation, and nutritional screening using tools such as the Mini Nutritional Assessment (MNA), reflecting a strong emphasis on evidence-based practice and health system frameworks. Similarly, Asia accounted for six studies (38%), emphasizing epidemiological and cohort analyses, cross-sectional assessments, and longitudinal tracking of intrinsic capacity, malnutrition, and functional transitions. Australia was represented by a single longitudinal cohort study (8%), providing valuable methodological insights and highlighting infrastructure for aging research. South Asia contributed one study (8%) that addressed health-related quality of life and linked clinical outcomes to broader population health measures. Notably, there is a paucity of research from Africa, South America, and other regions, revealing a significant gap in globally representative evidence. This uneven distribution underscores the need for international collaboration and harmonization of aging and oral health research, particularly within ICOPE-aligned frameworks, to ensure equitable, context-sensitive strategies for promoting intrinsic capacity, functional ability, and healthy aging worldwide.

Author Analysis

A total of 84 authors contributed to the 13 publications included in the dataset. All 84 authors are represented by only one publication each. The authorship country network highlights Switzerland as the central hub linking multiple international partners. Strong authorship ties exist between Switzerland and France, suggesting active European collaboration. Switzerland also connects with Australia and Thailand, indicating broader global participation. Australia and Thailand appear indirectly linked through Switzerland rather than collaborating directly. The color gradient suggests varying publication influence, with Switzerland and France showing moderate to higher contributions. Overall, the pattern demonstrates Switzerland’s key role in coordinating international authorship and strengthening cross-continental research cooperation.

**Figure 2:**
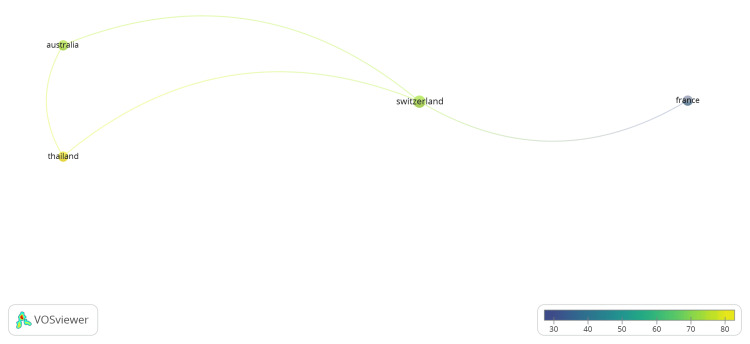
Switzerland serves as the central authorship hub, linking France, Australia, and Thailand, indicating strong collaboration and global research connectivity. VOSviewer (Leiden, The Netherlands: Centre for Science and Technology Studies, Leiden University)

**Figure 3:**
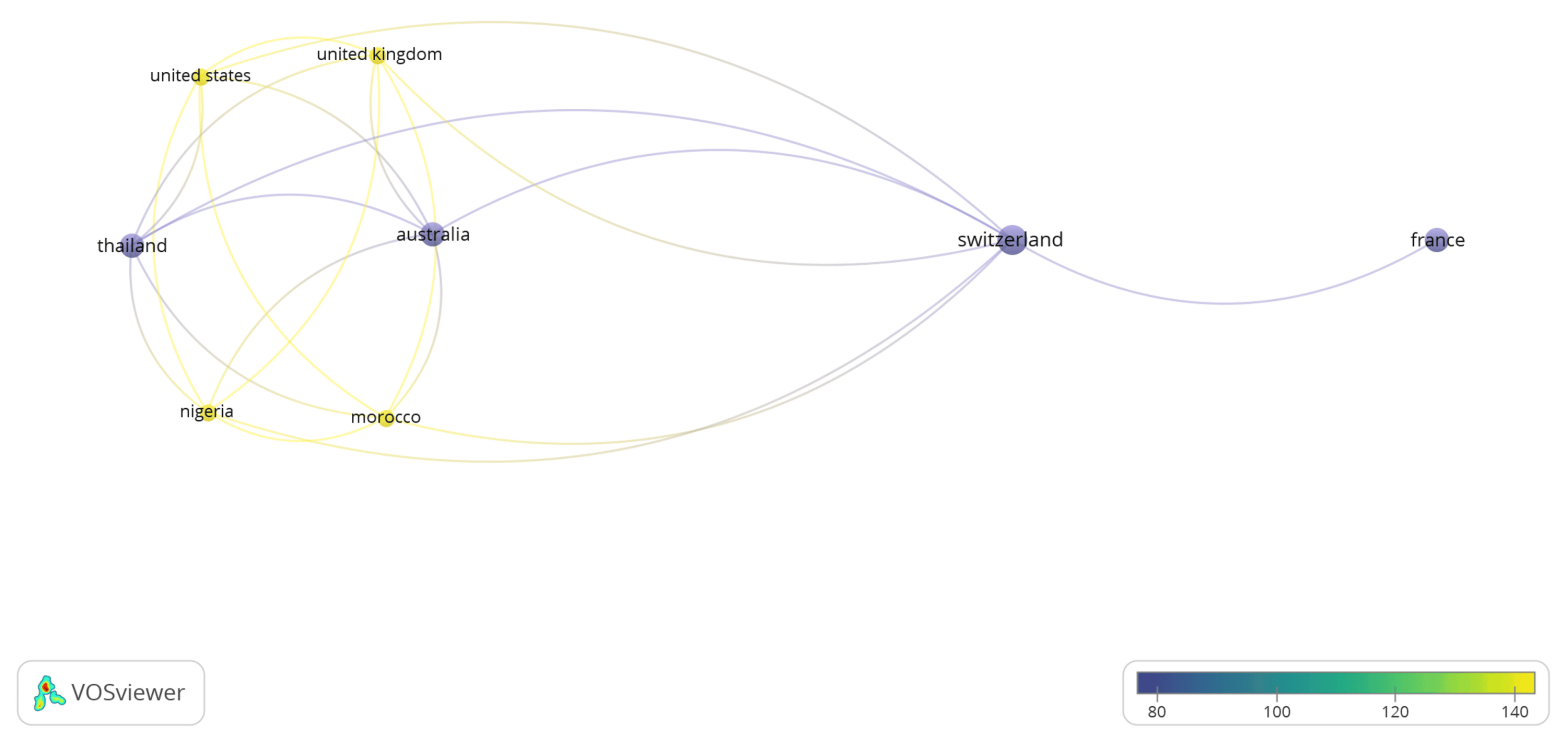
Coauthorship countries. The collaboration network for coauthorship analysis for countries based on average citation score, with Switzerland, and France received among the highly cited scores. VOSviewer (Leiden, The Netherlands: Centre for Science and Technology Studies, Leiden University)

The coauthorship network analysis reveals clear international collaboration patterns centered on Switzerland. Switzerland is the primary hub, connecting research partnerships with France, Australia, and Thailand. The strongest collaborative links are between Switzerland and France, indicating close regional cooperation within Europe. Switzerland also maintains notable connections with Australia and Thailand, reflecting broader intercontinental research engagement. Meanwhile, Australia and Thailand collaborate indirectly through Switzerland rather than directly, highlighting Switzerland’s mediating role in knowledge exchange. Overall, the network demonstrates Switzerland’s strategic position in fostering cross-regional collaboration and facilitating global research partnerships across Europe, Asia, and Australia (Figures 2, 3).

Bibliometric Exposure Window and Citation Density

**Figure 4:**
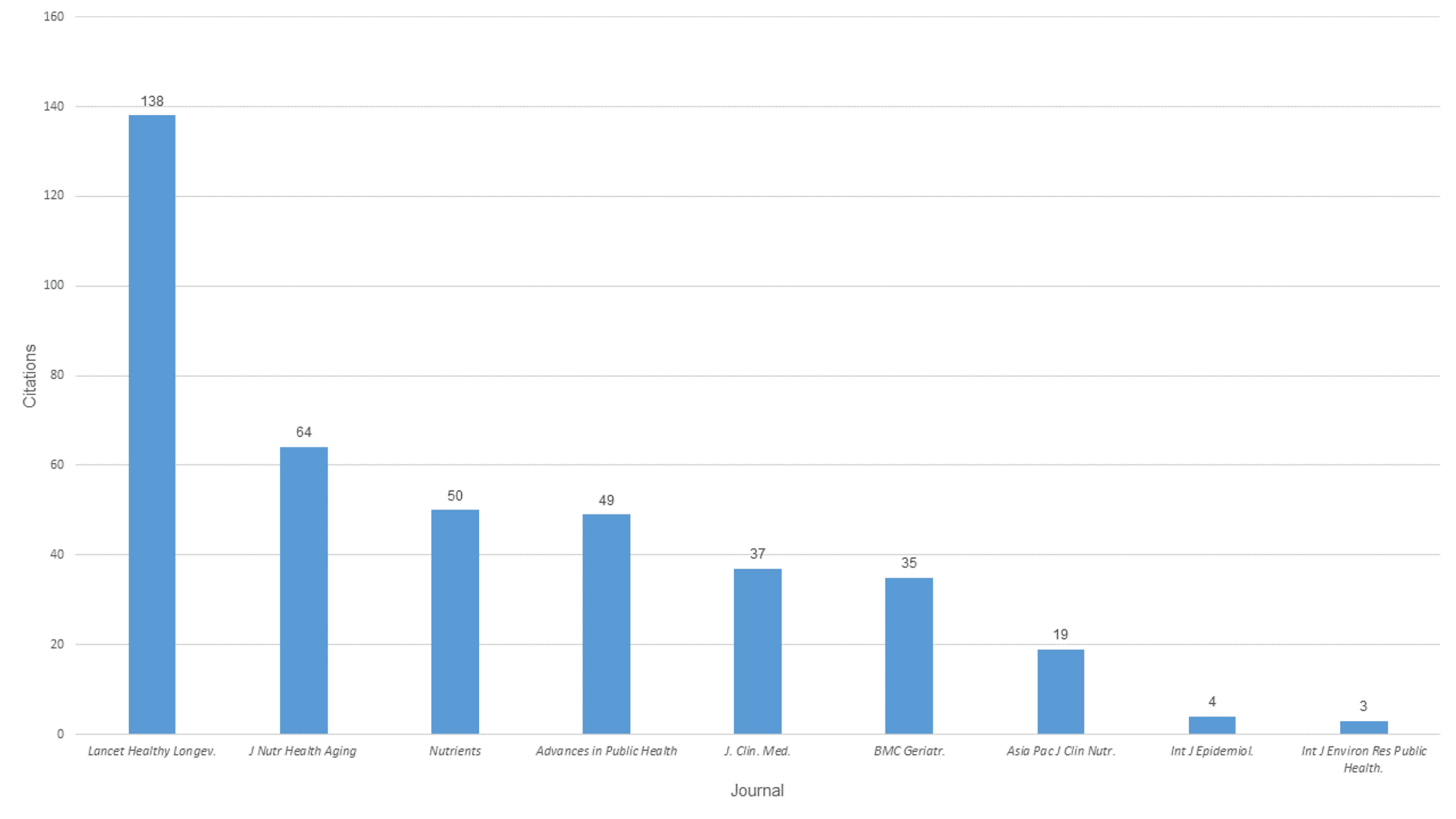
Number of citations by journal.

The bibliometric exposure window ranged from 0 to eight years. Woo et al. and Patel et al. had the highest cumulative citations due to longer exposure, with Woo et al. demonstrating significant influence on the conceptualization of geriatric oral health [9,10]. Citation density analysis allowed standardization across older and newer publications as follows: high citation densities were observed for foundational works [9,10], moderate for methodological or regional studies [12,14,18], and low for recent publications with short exposure windows [19,20,21]. The journal with the highest number of citations was the Lancet Healthy Longevity, with 138 citations, followed by Journal of Nutrition, Health and Aging with 64 citations and Nutrients with 50 citations (Figure 4).

Citation density indicates a marked rise in scholarly attention to aging-related health topics over time. Early activity was minimal, with only small citation counts recorded between 2018 and 2020, followed by gradual growth in 2021-2022. A sharp increase is evident from 2023 onward, peaking in 2025 with 195 citations, indicating accelerating impact and visibility of recent publications. The most influential paper, “Oral health for healthy aging (2021),” accumulated 138 citations and a high average citation per year (23.0), highlighting sustained relevance. Other high-density contributions address nutritional assessment, frailty, and quality of life, reflecting key geriatric priorities. Overall, citation patterns suggest expanding interdisciplinary interest and rapid knowledge consolidation in research on healthy aging, frailty, nutrition, and oral function (Table 3).

**Table 3:** Number of citations per year by document on ICOPE and removable prosthodontics in older adults (n=13). ICOPE: Integrated Care for Older People; NHANES: National Health and Nutrition Examination Survey

S. no.	Studies	Documents	Years	2018	2019	2020	2021	2022	2023	2024	2025	2026	Total	Average citation (AC) per year
1	Woo et al. (2018) [9]	Chewing difficulty should be included as a geriatric syndrome	2018	0	4	5	8	7	7	4	15	0	50	5.56
2	Patel et al. (2021) [10]	Oral health for healthy aging	2021	0	0	0	0	7	22	44	59	6	138	23.00
3	Ma et al. (2021) [11]	Frailty in China: from research to practice	2021	0	0	0	3	18	10	13	20	0	64	10.67
4	Guigoz and Vellas (2021) [12]	Nutritional assessment in older adults: MNA 25 years of a screening tool and a reference standard for care and research; what next?	2021	0	0	0	0	0	0	7	38	4	49	16.34
5	Khalatbari-Soltani et al. (2022) [13]	Cohort profile update: the Concord Health and Ageing in Men Project (CHAMP)	2022	0	0	0	0	0	0	5	30	2	37	12.34
6	Jia et al. (2023) [15]	Associations between transitions of intrinsic capacity and frailty status, and 3-year disability	2023	0	0	0	0	0	1	16	15	3	35	8.75
7	Chuansangeam et al. (2022) [14]	Prevalence and risk for malnutrition in older Thai people: a systematic review and meta-analysis	2022	0	0	0	0	0	3	8	7	1	19	3.80
8	Rony et al. (2024) [16]	Challenges and advancements in the health-related quality of life of older people	2024	0	0	0	1	3	3	3	8	0	18	3.00
9	Sciacchitano et al. (2024) [17]	To be frail or not to be frail: this is the question - a critical narrative review of frailty	2024	0	0	0	0	2	1	1	0	0	4	0.80
10	Eggimann et al. (2024) [18]	A comparison of oral function in older in- and out-patients: an observational study	2024	0	0	0	0	0	0	0	3	0	3	1.00
11	Dibello et al. (2025) [19]	Tooth loss as a risk factor of declining intrinsic capacity domains in later life: evidence from NHANES 2009-2014	2025	0	0	0	0	0	0	0	0	0	0	0.00
12	Rani et al. (2025) [20]	Challenges and recommendations for oral healthcare of older adults in a long-term care facility	2025	0	0	0	0	0	0	0	0	0	0	0.00
13	Scuttenaire and Braud (2026) [21]	Anti-resorptive therapy for osteoporosis and oral status of geriatric inpatients: a retrospective hospital-based study	2026	0	0	0	0	0	0	0	0	0	0	0.00
Total				0	4	5	12	37	47	101	195	16	417	-

Analysis of Author Keywords

**Figure 5:**
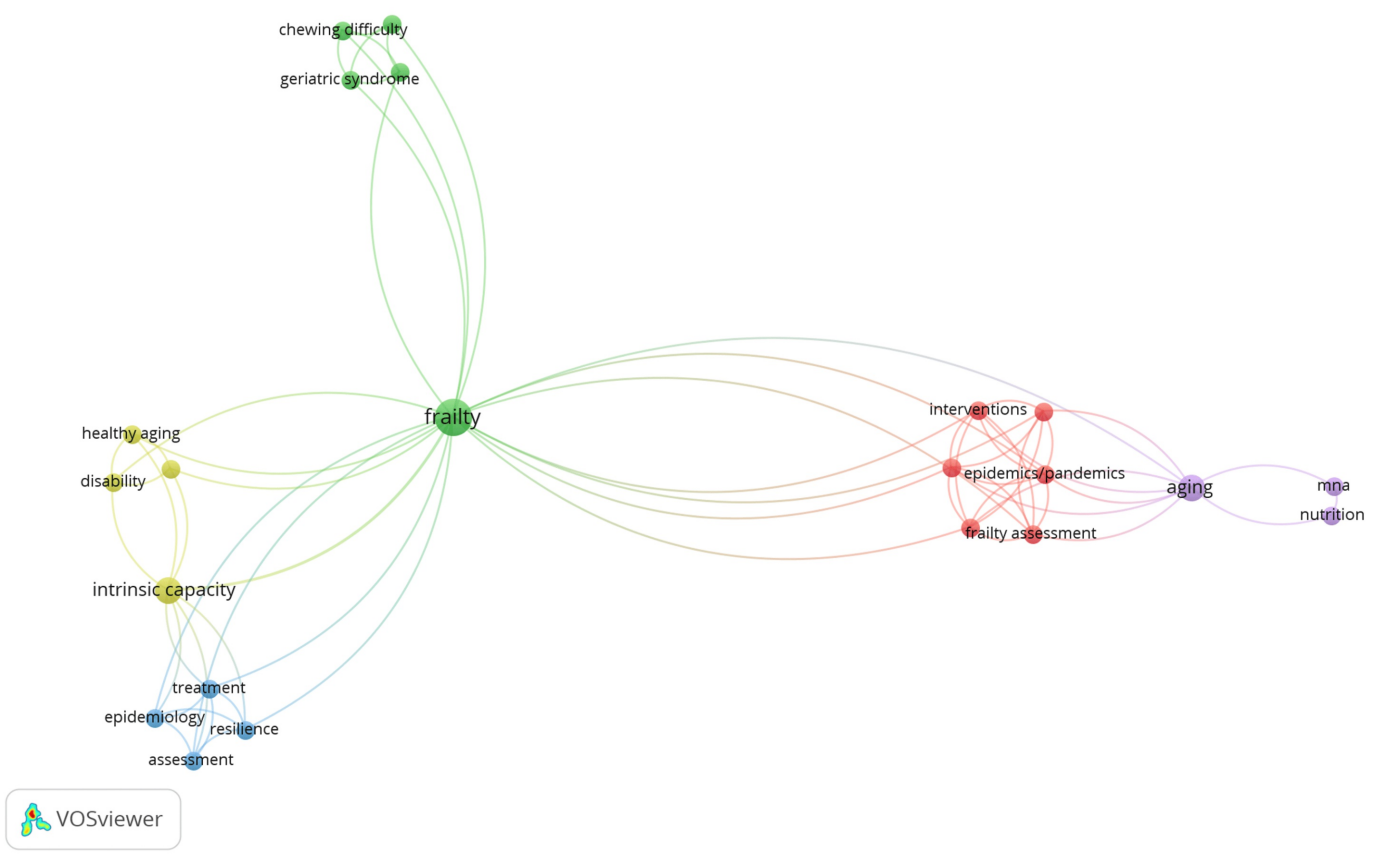
Larger nodes and denser connections indicate higher cooccurrence frequencies, highlighting key themes and their relationships. Network visualization of keyword occurrences based on the authors’ keywords (minimum number of occurrences of a keyword: 2), displaying 48 items grouped into five distinct clusters. VOSviewer (Leiden, The Netherlands: Centre for Science and Technology Studies, Leiden University)

The cooccurrence analysis of author keywords reveals frailty as the central and most interconnected concept, linking multiple thematic clusters. One cluster links frailty to geriatric syndrome and chewing difficulty, emphasizing clinical and functional health conditions in older adults. Another cluster associates frailty with intrinsic capacity, disability, and healthy aging, reflecting perspectives on functional ability and aging well. A third grouping links epidemiology, assessment, resilience, and treatment, highlighting measurement and public health approaches. Additionally, frailty connects to epidemics/pandemics, interventions, and frailty assessment, indicating growing interest in health system responses and risk management. Finally, a smaller cluster related to aging, nutrition, and MNA underscores the importance of nutritional assessment in older populations. Overall, the network shows frailty as a multidisciplinary concept bridging clinical care, public health, and healthy aging frameworks (Figure 5).

Oral Health, Frailty, and Intrinsic Capacity Integration

The majority of studies demonstrate that oral health status, including functional decline and tooth loss, is closely associated with transitions in frailty and intrinsic capacity domains. Woo et al. first described chewing difficulty as a geriatric syndrome associated with declines in mobility and vitality [9]. Dibello et al. extended this evidence, showing that tooth loss predicts declines in intrinsic capacity [19]. Eggimann et al. compared oral function between inpatients and outpatients, confirming the impact of care setting on oral hypofunction [18]. Frailty-focused reviews and cohort analyses reinforced these links and provided longitudinal insights [11,15,17]. Policy and systems-level studies highlighted the importance of integrating oral health into healthy aging frameworks [10,20].

A distinct nutrition-focused subcluster emerged from Guigoz and Vellas and Chuansangeam et al., highlighting malnutrition prevalence, frailty risk, and functional decline in older adults [12,14]. These studies demonstrated that nutritional deficits are closely linked to oral health outcomes, particularly chewing difficulty and tooth loss, which in turn mediate sarcopenia and decline in intrinsic capacity. Guigoz and Vellas synthesized 25 years of Mini Nutritional Assessment (MNA) data, emphasizing its utility in screening, frailty prediction, and integration into clinical practice [12]. Chuansangeam et al. provided epidemiological evidence among Thai older adults, reporting a high prevalence of malnutrition and quantifying associated risk factors [14]. Together, these studies form the nutrition-frailty-oral health axis, a key mechanistic pathway within the healthy aging framework.

Conceptual Pathway

By synthesizing evidence across the included studies, a refined conceptual pathway can be delineated (Table 4). Oral health, particularly masticatory function and prosthodontic rehabilitation, directly influences nutritional status, as measured by tools such as the MNA. Adequate nutrition, in turn, supports the preservation of muscle mass and function, mitigating sarcopenia and declines in intrinsic capacity. Impairments in these domains contribute to frailty transitions, thereby increasing the risk of disability and compromising health-related quality of life (HRQoL). The cumulative impact of these factors underscores the importance of integrating oral health and nutritional interventions within broader healthy aging frameworks and informs evidence-based policy development. This model highlights the multidimensional interplay between clinical, functional, and policy-level determinants of healthy aging, offering a structured approach to guide research priorities, clinical assessment, and targeted interventions for older adults. Table 4 presents a conceptual framework derived from the bibliometric clusters and qualitative synthesis of included studies; it is interpretive rather than a direct empirical output.

**Table 4:** Conceptual clusters linking oral health, nutrition, intrinsic capacity, frailty, and quality of life in older adults and their contributions to the WHO ICOPE framework. OHRQoL: oral health-related quality of life; HRQoL: health-related quality of life; ICOPE: Integrated Care for Older People; IC: intrinsic capacity; LTC: long-term care

Cluster	Conceptual domain	Key themes	Representative evidence	Contribution to ICOPE framework
Cluster 1	ICOPE and intrinsic capacity (core)	Intrinsic capacity domains, early functional decline, nutrition, mobility, psychological and sensory function	Beard et al. (2016) [1]; Cesari et al. (2018) [2]; WHO (2019) a and b [3,4]; WHO 2024 [5]; Woo et al. (2018) [9]; Ma et al. (2021) [11]; Jia et al. (2023) [15]; Sanchez-Rodriguez et al. (2021) [22]	Defines intrinsic capacity domains and supports early detection and monitoring of functional decline central to ICOPE implementation
Cluster 2	Oral function and frailty (bridging)	Oral hypofunction, tooth loss, frailty progression, LTC barriers, and IC decline associations	Miyahara et al. (2024) [7]; Sciacchitano et al. (2024) [17]; Eggimann et al. (2024) [18]; Dibello et al. (2025) [19]	Demonstrates how oral function indicators can signal IC decline and frailty risk, supporting integrated functional assessments
Cluster 3	Removable prosthodontics and quality of life	Dentures, masticatory function, nutrition (MNA), OHRQoL, cognitive outcomes, policy integration	Yen et al. (2015) [6]; Ahmed et al. (2021) [8]; Patel et al. (2021) [10]; Guigoz and Vellas (2021) [12]; Rony et al. (2024) [16]	Provides actionable interventions that improve nutrition, function, and participation, helping maintain or restore intrinsic capacity
Cluster 4	Cognitive and psychosocial outcomes (emerging)	Social participation, psychological well-being, HRQoL, cognitive function	WHO (2024) [5]; Woo et al. (2018) [9]; Patel et al. (2021) [10]; Khalatbari-Soltani et al. (2022) [13]; Rony et al. (2024) [16]; Dibello et al. (2025) [19]; Rani et al. (2025) [20]	Extends ICOPE beyond physical domains by supporting psychological well-being, social engagement, and cognitive health

Discussion

Scientometrics analysis is an essential tool for evaluating scientific research output and understanding trends within academic fields [22,23]. Hirsch introduced the H-index as a metric to quantify an individual researcher’s scientific contributions, balancing productivity and citation impact [24,25]. Building on traditional citation-based metrics, Roldan-Valadez et al. discussed a range of scientometric indicators, including the impact factor and alternative metrics, and highlighted their applications and limitations for assessing both journals and researchers [26]. Clarivate Analytics found that journal self-citation can significantly influence these metrics, potentially inflating perceived impact if not properly accounted for [27]. Kokol et al. explored how bibliometrics can be applied in medicine, showing how historical analyses can reveal research trends, productivity patterns, and the evolution of scientific focus areas, emphasizing the value of bibliometric tools for both evaluation and strategic research planning [28].

This scientometric and narrative synthesis integrates 13 studies published between 2018 and January 31, 2026, examining oral health, nutrition, intrinsic capacity (IC), frailty, and quality of life in older adults. Table 4 presents a conceptual framework, not a direct empirical finding. The four domains were partially data-driven, based on VOSviewer bibliometric clustering and thematic patterns in the included studies. The pathway and directional links were constructed post-hoc through qualitative synthesis, integrating observational associations, mechanistic evidence, and WHO ICOPE principles. This framework is hypothesis-generating, illustrating potential connections among oral function, nutrition, intrinsic capacity, frailty, and quality of life, while clearly distinguishing data-driven patterns from the author's interpretation.

Using a cluster-based approach, the literature can be grouped into the following four conceptual domains: Cluster 1 (ICOPE and intrinsic capacity {core}), Cluster 2 (oral function and frailty {bridging}), Cluster 3 (removable prosthodontics and quality of life), and Cluster 4 (cognitive and psychosocial outcomes {emerging}). European and Asian studies dominate the field, each contributing five studies, while Australia, Asia, and non-indexed studies provide unique longitudinal, HRQoL, and pharmacotherapy perspectives. European studies emphasize oral function, nutrition, frailty assessment, and policy integration [10,12,17-19], whereas Asian studies focus on malnutrition, intrinsic capacity transitions, oral hypofunction, and functional decline [9,11,14,15,20].

Cluster 1 (ICOPE and Intrinsic Capacity)

The WHO ICOPE model forms the theoretical and empirical backbone of the framework [3-5]. It emphasizes intrinsic capacity domains-vitality (nutrition), mobility, psychological well-being, sensory function, and functional ability, as central to healthy aging. Empirical applications of ICOPE demonstrate its utility in capturing functional decline in community-dwelling older adults [28,29]. For example, Woo et al. highlighted chewing difficulty as a prevalent geriatric syndrome strongly associated with frailty, aligning with ICOPE’s focus on early detection of functional impairments [9]. Similarly, Ma et al. and Jia et al. traced IC transitions and frailty trajectories in Chinese older adults, illustrating how oral function and nutritional status serve as measurable indicators of broader IC decline [11,15]. These studies demonstrate the relevance of ICOPE-aligned assessments to oral health and frailty outcomes, emphasizing a systems-level approach to healthy aging [1,2].

Cluster 2 (Oral Function and Frailty)

It serves as a conceptual bridge between IC and clinical oral health outcomes. Eggimann et al. compared oral hypofunction between hospitalized and community-dwelling older adults, revealing substantial variability in oral functional decline [18]. Dibello et al. reported associations between tooth loss and declines in intrinsic capacity, highlighting oral status as a potential predictor of functional outcomes [19]. Rani et al. identified long-term care (LTC) barriers to oral health in Malaysia, linking systemic and environmental factors to frailty progression [20]. Miyahara et al.’s evidence that oral hypofunction correlates with IC decline, which aligns with Sciacchitano et al.'s contextualized oral function within frailty models, contrasting phenotype and deficit accumulation frameworks [7,17]. Associations between self-reported oral health and multiple IC domains, including locomotion and vitality, reinforce the importance of assessing oral function as part of holistic IC evaluations.

Cluster 3 (Removable Prosthodontics and Quality of Life)

Prosthetic rehabilitation is underscored as an actionable intervention influencing IC domains. Guigoz and Vellas demonstrated that the Mini Nutritional Assessment (MNA) is a validated tool that links malnutrition, sarcopenia, and frailty, providing a mechanistic pathway by which prosthodontic interventions can influence functional outcomes [12]. Yen et al. showed that removable dentures improve oral health-related quality of life (OHRQoL) and functional ability, supporting their integration into IC-enhancing interventions [6]. Ahmed et al. extended this evidence to cognitive function, suggesting that prosthetic rehabilitation may modulate higher-order IC domains, complementing WHO ICOPE’s multidimensional approach [8]. Patel et al. reinforced the policy relevance by advocating for oral health inclusion in European healthy aging frameworks, while Rony et al. highlighted determinants of HRQoL in Bangladesh, demonstrating that oral interventions influence psychosocial and functional outcomes [10,16].

Cluster 4 (Cognitive and Psychosocial Outcomes)

Although emerging, the literature highlights the broader benefits of oral health interventions. Studies indicate that oral function, prosthodontic rehabilitation, and nutritional status affect social participation, psychological well-being, and HRQoL [9,10,16]. These findings align with ICOPE’s comprehensive approach to maintaining intrinsic capacity and functional ability across multiple domains, demonstrating the synergistic potential of oral and nutritional interventions in supporting healthy aging.

Geographically, Europe and Asia dominate the literature, suggesting regional research strengths and funding availability, while Australia and Asia contribute unique longitudinal and HRQoL perspectives [13,16]. Bibliometric indicators highlight early recognition of oral function and frailty [9,10], and a recent surge in research linking nutrition, IC, and prosthodontics [19,20]. This pattern underscores a growing interest in integrating oral health into ICOPE-aligned frameworks, particularly post-2020, coinciding with the global implementation of the ICOPE strategy [5].

The currently available body of evidence, although limited and methodologically heterogeneous, suggests an association between oral health status, particularly functional decline and tooth loss, and trajectories of frailty and intrinsic capacity. However, this relationship is predominantly supported by observational and conceptual studies rather than robust causal evidence. The literature comprises a mixture of narrative reviews, cross-sectional analyses, and a relatively small number of longitudinal investigations, thereby constraining definitive inference. Earlier longitudinal studies did not consistently demonstrate a clear association between poor oral health and incident frailty [30], whereas more recent studies indicate a potential relationship between tooth number, masticatory function, and frailty status [31-33]. In addition, a biologically plausible framework is supported by the concept of inflammaging, whereby chronic low-grade inflammation contributes to both systemic and oral functional decline [34-36]. Within this context, oral functional impairments, including reduced chewing ability and tooth loss, appear to be associated with broader frailty phenotypes; nevertheless, the evidence remains largely associative, and there is a notable lack of longitudinal and interventional studies directly addressing intrinsic capacity domains or prosthodontic outcomes [37].

Synthesizing these findings supports a refined conceptual pathway as follows: oral health influences mastication and nutrition (MNA), which in turn mediates sarcopenia and IC decline, thereby precipitating frailty, disability, and reductions in HRQoL, ultimately informing healthy aging policy and integrated care models (Table 4). Prosthodontic interventions, such as removable dentures, represent clinically actionable components within this pathway and have the potential to improve nutritional intake, masticatory function, psychosocial engagement, and possibly cognitive outcomes. In addition, psychological dimensions of care, including patient attitudes and perceived satisfaction with removable complete dentures, appear to play a significant role in treatment outcomes [38]. However, despite this conceptual alignment, oral health and prosthodontic care remain underrepresented in existing ICOPE assessments, highlighting an important gap in both research and policy [3-5]. Oral hypofunction is a condition characterized by a decline in multiple oral functions, including mastication, swallowing, salivation, tongue-lip motor function, and oral hygiene status, often diagnosed when several functional impairments coexist [39]. Removable prosthodontics is the branch of prosthodontics concerned with the replacement of missing teeth and associated structures using prostheses that can be removed and reinserted by the patient [40].

In conclusion, these scientometric clusters and empirical findings underscore the multidimensional relationship among oral health, nutrition, IC, frailty, and quality of life. Integrating oral function and prosthodontic rehabilitation into ICOPE-aligned frameworks can enhance early detection of functional decline, optimize nutritional status, sustain intrinsic capacity, and support psychosocial well-being in older adults. Future research should prioritize longitudinal and interventional studies that explicitly embed prosthodontic care within ICOPE assessments, strengthening the evidence base for clinical practice and health policy aimed at promoting functional independence and healthy aging. This review focuses specifically on removable prosthodontic rehabilitation as a clinical intervention aimed at restoring oral function in partially or fully edentulous older adults. While oral health refers broadly to the overall state of the oral cavity, and oral function denotes the physiological capacity for mastication, swallowing, and speech, the scope here is on how prosthodontic treatment may support oral function and, in turn, influence nutrition, intrinsic capacity, frailty, and quality of life within the WHO ICOPE framework. Distinguishing these concepts provides a focused synthesis of evidence linking prosthodontic interventions to healthy aging outcomes.

## Conclusions

This scientometrics study highlights the multidimensional interplay among oral health, IC, frailty, and healthy aging within the WHO ICOPE framework. European and Asian studies dominate the literature, with key contributions emphasizing clinical assessments, prosthodontic interventions, nutritional status, and longitudinal monitoring of IC trajectories. Evidence shows that oral hypofunction, tooth loss, and reduced masticatory ability are significantly associated with declines in IC domains, frailty progression, and reductions in health-related quality of life (HRQoL). Removable prosthodontic rehabilitation, including complete and partial dentures, emerges as a clinically actionable intervention that improves oral function, nutritional intake, psychosocial well-being, and cognitive outcomes, thereby influencing multiple dimensions of intrinsic capacity. Cluster analysis identifies four conceptual domains as follows: ICOPE and IC; oral function and frailty; removable prosthodontics and quality of life; and cognitive and psychosocial outcomes, highlighting the current fragmentation of research and the underrepresentation of oral health in ICOPE assessments. Integrating oral function and prosthodontic care into ICOPE-aligned frameworks could enhance early detection of functional decline, support maintenance of IC, mitigate frailty, and promote overall healthy aging. Future research should prioritize longitudinal and interventional studies that embed prosthodontic rehabilitation within ICOPE assessments, foster interdisciplinary approaches, and inform evidence-based clinical practice and policy development for older adults.
